# Tourniquet use during total knee arthroplasty does not modulate the neutrophil-to-lymphocyte ratio, pain, or activity

**DOI:** 10.1007/s10195-016-0435-6

**Published:** 2016-11-15

**Authors:** Tyler Barker, Victoria E. Rogers, Kimberly B. Brown, Vanessa T. Henriksen, G. Lynn Rasmussen

**Affiliations:** 1 The Orthopedic Specialty Hospital, 5848 S. Fashion Blvd., Murray, UT 84107 USA; 2 The Orthopedic Specialty Clinic, Murray, UT 84107 USA

**Keywords:** Neutrophil-to-lymphocyte ratio, Total knee arthroplasty, Pain, Activity

## Abstract

**Abstract:**

The purpose of our study was to identify the influence of tourniquet use during total knee arthroplasty (TKA) on the neutrophil-to-lymphocyte ratio (NLR) shortly after surgery and patient-reported outcomes (pain and physical activity) from outpatient physical therapy. This retrospective study consisted of 104 subjects who underwent primary unilateral TKA (51 subjects with and 53 subjects without tourniquet assistance) between 2010 and 2012. The NLR was calculated from the absolute neutrophil and lymphocyte counts obtained immediately before and after (1 and 2 days) knee arthroplasty. The Knee Outcome Survey (KOS) of Activities of Daily Living and numeric pain scores collected at the first [33.0 (34.2) days after surgery] and last [85.5 (40.7) days after surgery] outpatient physical therapy visits were extracted from an electronic database. The NLR, pain, and KOS score were not significantly (all *p* > 0.05) different with tourniquet use. Based on these findings, we conclude that tourniquet use during TKA neither increases systemic inflammation shortly after surgery nor impairs patient-reported outcomes obtained during outpatient physical therapy.

**Level of evidence:**

IV.

## Introduction

Tourniquet use during total knee arthroplasty (TKA) improves acuity by creating a bloodless operating field. However, tourniquet inflation and deflation creates an ischemia–reperfusion response that increases systemic inflammation [1]. Inflammation is essential for host defense and repair processes, but an exaggerated inflammatory response can be detrimental to a variety of physiological systems, including musculoskeletal, immune, and cardiovascular. In recent years, the neutrophil-to-lymphocyte ratio (NLR) has emerged as a sensitive index of systemic inflammation that predicts serious complications, such as pulmonary embolism or cardiovascular disease risk [2, 3]. Deviations in the NLR are also apparent with contrasting arthroplasty procedures (unicompartmental versus tricompartmental) [4], suggesting that the NLR could serve as an indicator for systemic inflammation and for the severity of surgery-induced trauma. Surprisingly, it is unknown if tourniquet use during TKA alters the NLR.

Tourniquet use also increases pain and physical impairment in the days after TKA [5], but it is unknown if tourniquet use impairs outpatient physical rehabilitation in the months after TKA. The purpose of our study was to identify the influence of tourniquet use during TKA on the NLR and patient-reported outcomes from outpatient physical therapy. We hypothesized that tourniquet-assisted TKA would exacerbate the increase in the NLR shortly after surgery and impair patient-reported outcomes (pain and physical function) during outpatient physical therapy.

## Materials and methods

Patients who underwent primary unilateral TKA were retrospectively identified using the International Classification of Diseases, 9th Revision code and limited to surgeries performed at one facility (The Orthopedic Specialty Hospital, Murray, UT USA) by one orthopedic surgeon between 2010 and 2012 (*n* = 462). This time frame was selected because half-way through 2011 the participating surgeon discontinued tourniquet use during TKA without altering other standard of care practices. For the purpose of limiting guideline and practice variability, we also excluded (*n* = 358) subjects who performed outpatient physical therapy at a separate facility. The final analysis consisted of 104 subjects—51 subjects with and 53 subjects without tourniquet assistance during TKA. Postoperative complications were documented for each subject. This study was approved by the Institutional Review Board at Intermountain Healthcare (Salt Lake City, UT USA).

On the day of surgery, the subjects were taken to the operating room and placed on the table in a supine position while a femoral nerve block was administered. Following administration, the subjects were assisted to a sitting position while spinal anesthetic was provided. The subjects were returned to a supine position and the involved limb was prepared and draped, after which prophylactic antibiotics were administered. For the tourniquet group, the leg was exsanguinated with an Esmarch bandage, and the tourniquet was placed as high as possible on the thigh of the surgical leg, over cast padding, and inflated to 300 mmHg.

Subjects underwent a tricompartmental knee arthroplasty through a medial parapatellar arthrotomy approach with uncemented fixation. After surgery, the tourniquet was deflated (when a tourniquet was used), and following wound closure, a Fresenius Cell Saver (Redmond, WA, USA) Hemovac^®^ drain was put in place in the joint. The medial parapatellar arthrotomy was initially closed using sutures followed by staples or sutures to close subcutaneous tissues and skin in layers. Adaptic and a sterile dressing were applied to the wounds. Subjects were then transferred to the recovery room. In the post-anesthesia care unit, subjects from both groups received patient-controlled analgesia with hydromorphone through a continuous ambulatory delivery device. The Fresenius Cell Saver system drains were removed on the first day after surgery. Tranexamic acid was not used in any subject and no subjects received a reinfusion after TKA.

The NLR was calculated from the absolute neutrophil and lymphocyte counts obtained immediately before (Pre) and after (1 and 2 days) TKA. Outpatient physical rehabilitation was initiated 33 (34.2) days after surgery. The Knee Outcome Survey (KOS) of Activities of Daily Living and numeric pain rating scale (0–10 for current pain) scores from the first and last physical therapy visits [52.5 (25.8) days between visits] and number of physical therapy visits [11.4 (5.9)] were extracted from an electronic database. Subjects were classified as responders if their KOS score increased by ≥11 points [6] or if their pain score decreased by ≥2 points [7].

Data were checked for normality prior to all statistical analyses with the Shapiro–Wilk test. Subject differences between tourniquet groups were assessed using a two-sample *t* test with a Bonferroni adjusted *p* value. Categorical variable differences between groups were evaluated using separate Pearson chi-squared tests. Blood chemistry and outpatient physical rehabilitation outcome differences between groups were assessed with separate repeated measures analysis of variance followed by a Bonferroni correction on multiple pairwise comparisons when appropriate. All statistical analyses were performed with SYSTAT (version 13.1, Chicago, IL USA). Data are presented as mean (SD) unless noted otherwise.

## Results

Subject characteristics, preoperative blood chemistries, American Society of Anesthesiologists (ASA) score, surgery time, length of stay, and postoperative complications were not significantly different between tourniquet groups (Tables 1 and 2; all *p* > 0.05). Tourniquet use during TKA did not magnify surgery-induced alterations in white blood cell count (WBC), neutrophil or lymphocyte absolute counts, the NLR, hemoglobin, or hematocrit levels (Table 2; all *p* > 0.05). Likewise, pain and KOS scores were not significantly different between groups (*p* value range 0.20−0.98).

**Table 1:** Subject and general surgery characteristics

	T−	T+	*p* value
*n* (f/m)	53 (28/25)	51 (33/18)	0.22
Age, years	62 (10)	63 (13)	0.81
Height, cm	170 (11)	170 (12)	0.99
Body mass, kg	96.2 (24.7)	99.3 (25.8)	0.53
Body mass index, kg/m^2^	33.2 (7.3)	34.4 (8.0)	0.42
ASA score, n*			0.13
2	5	1	
3	23	17	
4	24	30	
Knee, left/right	23/30	27/24	0.33
Surgery time, min	125 (17)	121 (18)	0.28
Tourniquet, min	NA	73.6 (23.5)	NA
Length of stay, days	3.04 (0.39)	2.96 (0.28)	0.25
Infections, *n*	1	0	0.32
Allogeneic transfusions, *n*	0	2	0.15
Manipulations under anesthesia, *n*	4	9	0.12
Pulmonary embolisms, *n*	0	0	NA
Deep venous thromboembolism, *n*	0	0	NA
Readmissions, n**	4	1	0.18

Data presented as mean (SD) unless noted otherwise. *T−* non-tourniquet-assisted TKA, *T+* tourniquet-assisted TKA, *NA* not applicable

* ASA score was missing for 1 T− subject and for 3 T+ subjects

** Reasons for readmission include abnormal serum enzyme level, tachycardia, other brain condition, atrial fibrillation, other esophagitis

**Table 2:** Perioperative blood chemistries and outpatient physical therapy data

	T−	T+	Tourniquet main effect *p* value	Time main effect *p* value	Interaction *p* value	Time-pairwise comparison *p*-values		
						Pre vs 1 day	Pre vs 2 days	1-day vs 2 days
Hemoglobin, g/dL			0.47	<0.001	0.10	<0.001	<0.001	<0.001
Pre	14.0 (1.5)	14.0 (1.6)						
1-day post	12.0 (1.5)	12.2 (1.4)						
2-day post	11.0 (1.3)	11.3 (1.3)						
Hematocrit, %			0.77	<0.001	0.78	<0.001	<0.001	<0.001
Pre	40.9 (5.6)	40.9 (6.5)						
1-day post	35.6 (4.3)	35.7 (4.1)						
2-day Post	32.7 (3.7)	33.1 (3.8)						
WBC, K/uL			0.15	<0.001	0.50	<0.001	<0.001	<0.001
Pre	7.34 (2.42)	6.60 (1.50)						
1-day post	11.2 (3.0)	10.3 (2.8)						
2-day post	9.63 (2.33)	9.22 (2.00)						
Neutrophils, K/uL			0.38	<0.001	0.47	<0.001	<0.001	<0.001
Pre	4.37 (1.34)	4.04 (1.24)						
1-day post	8.75 (2.31)	8.21 (2.64)						
2-day post	6.78 (1.59)	6.72 (1.65)						
Lymphocytes, K/uL			0.58	<0.001	0.27	<0.001	0.004	0.001
Pre	2.06 (1.26)	1.88 (0.57)						
1-day post Post	1.38 (1.42)	1.17 (0.54)						
2-day post Post	1.64 (1.23)	1.72 (1.08)						
NLR			0.44	<0.001	0.80	<0.001	<0.001	<0.001
Pre	2.40 (0.89)	2.35 (1.06)						
1-day post	8.62 (5.00)	8.19 (4.07)						
2-day post	5.41 (3.32)	4.82 (2.35)						
∆1	6.23 (4.96)	5.78 (3.88)	0.61					
∆2	2.99 (3.16)	2.44 (2.04)	0.29					
∆1-2	−3.23 (5.40)	−3.34 (3.79)	0.91					
Pain			0.20	<0.001	0.46			
First PT visit	5.22 (2.24)	4.79 (2.23)						
Last PT visit	3.28 (2.67)	2.42 (1.82)						
Pain ∆	−2.42 (2.09)	−1.94 (3.10)	0.36					
Pain responder	26	33	0.24					
KOS			0.82	<0.001	0.87			
First PT visit	46.7 (17.1)	47.9 (16.9)						
Last PT visit	68.6 (18.4)	69.9 (17.0)						
KOS ∆	21.9 (16.3)	21.8 (19.3)	0.98					
KOS responder	36	42	0.31					

Data presented as mean (SD)

*T*− non-tourniquet-assisted TKA, *T*+ tourniquet-assisted TKA, *PT* physical therapy, *WBC* white blood cell, ∆*1* change from Pre to 1 day, ∆*2* change from Pre to 2 days, ∆*1-2* change from 1 day to 2 day

## Discussion

In contrast to our hypothesis, the increase in the NLR was not modulated by tourniquet use during TKA. This finding is consistent with a previous study demonstrating that tourniquet-assisted TKA did not exacerbate the systemic increase in C-reactive protein following knee arthroplasty [8]. However, the findings by Matziolis et al. [8] and those in the present study could be limited by protocol design as the earliest postoperative assessments of systemic inflammation were performed 1 day after surgery. Consistent with this perceived limitation, data demonstrate an exaggerated inflammatory response within 24 h after tourniquet-assisted TKA [1]. Thus, the systemic inflammatory response to tourniquet-assisted TKA could be temporally dependent with deviations likely apparent within the first 24 h after surgery.

Similar to the NLR, pain levels reported during outpatient physical therapy were not altered by tourniquet use during TKA. This finding contrasts with an earlier report showing tourniquet use during TKA increases postoperative pain shortly (4 h) after knee arthroplasty [9]. Considering the present study’s first postoperative pain assessment was obtained ~33 days after surgery, it is plausible that tourniquet-induced pain is more readily apparent within hours but not days after TKA.

Data also indicate that tourniquet assistance during TKA impedes progression in an objective-based measure of physical performance, such as muscle strength and knee range of motion [9]. However, in the present investigation, tourniquet use did not alter patient-reported physical activity levels during outpatient physical therapy. While improvements in subjective-based outcomes exceed those of more objectively routed measures following TKA [10], it remains unknown if tourniquet use during TKA contributes to the discordance of findings between subjective and objective outcomes after surgery. Clearly, future research investigating the role of tourniquet use on subjective- and objective-based physical performance outcomes following TKA is warranted.

There are some limitations with this study that are worthy of discussion. First, besides the NLR, no other biomarkers of systemic inflammation were examined. However, previous research performed in non-orthopedic patients (i.e., cardiovascular and colorectal cancer) demonstrates that the NLR associates with other common indices of systemic inflammation, such as C-reactive protein and interleukin-6 [11, 12]. Second, including blood clotting, range of motion, and objective-based outcomes would strengthen the evaluation of tourniquet use during knee arthroplasty. Future research is needed to identify the association between the NLR and other systemic indices of inflammation and objective-based outcomes following TKA.

In summary, tourniquet use during TKA did not increase systemic inflammation or hinder patient-reported outcomes. Based on these findings, we conclude that tourniquet use during TKA neither increases systemic inflammation nor is detrimental to patient-reported outcomes during physical therapy.
